# A Case of Three Synchronous Primary Lung Cancers of Distinct Histologies in a Patient With Severe Chronic Obstructive Pulmonary Disease

**DOI:** 10.7759/cureus.104605

**Published:** 2026-03-03

**Authors:** Matthew T Foley, Kyle A Burton, Sheetal Acharya, Brian Pahn, Matthew Karulf

**Affiliations:** 1 Internal Medicine, Michigan State University College of Human Medicine, East Lansing, USA; 2 Medicine, University of Wisconsin, Madison, USA; 3 Hematology and Medical Oncology, Upper Peninsula Health System, Marquette, USA; 4 Pathology, Upper Peninsula Health System, Marquette, USA; 5 Pulmonology, Upper Peninsula Health System, Marquette, USA

**Keywords:** chronic obstructive pulmonary disease (copd), copd, lung adenocarcinoma, non-small cell lung cancer, respiratory oncology, small cell lung cancer, squamous cell carcinoma (scc), synchronous primary cancers, triple synchronous lung cancer

## Abstract

Synchronous primary lung cancers are uncommon and present significant diagnostic and therapeutic challenges, particularly when tumors of differing histologies are identified concurrently. Multiple primary lung cancer (MPLC) is defined as the occurrence of two or more distinct primary malignant tumors within the lungs of a single patient, either synchronously or metachronously. Synchronous MPLC requires careful differentiation from intrapulmonary metastases, as misclassification may significantly alter staging and treatment intent. Triple synchronous MPLC is exceptionally rare and is described primarily in isolated case reports and small case series. This case report describes a 65-year-old woman with severe chronic obstructive pulmonary disease (COPD) who was found to have three synchronous primary lung cancers, including limited-stage small cell lung cancer and two distinct non-small cell lung cancers, following evaluation of new pulmonary nodules on surveillance imaging. Comprehensive histopathologic evaluation and multidisciplinary decision-making guided curative-intent concurrent chemoradiation, highlighting the importance of thorough diagnostic sampling and individualized treatment planning in complex presentations. The patient completed therapy but died approximately three months after diagnosis from complications related to advanced COPD and lung malignancy, underscoring the competing mortality risks in this population. This case emphasizes that accurate histologic confirmation of each lesion is essential to ensure appropriate staging under the eighth edition of the tumor, node, metastasis (TNM) classification system and to distinguish synchronous primaries from metastatic disease. Furthermore, coordinated multidisciplinary collaboration among pulmonology, pathology, medical oncology, and radiation oncology is critical to balance oncologic control with preservation of functional status, particularly in patients with severe baseline pulmonary impairment.

## Introduction

Multiple primary lung cancer (MPLC) is defined as the occurrence of two or more primary malignant lung tumors in a single patient and may present as either synchronous or metachronous disease. Synchronous MPLC describes multiple primary lung cancers identified simultaneously, while metachronous MPLC refers to the development of a new primary lung cancer following a tumor-free interval after treatment of an initial malignancy [1]. Although historically considered rare, the reported incidence of MPLC has increased with advances in computed tomography (CT) and positron emission tomography (PET), which have improved the detection of distinct pulmonary lesions [2].

Patients with significant tobacco exposure, chronic obstructive pulmonary disease (COPD), and prior lung malignancy are at increased risk for developing MPLC. Differentiating synchronous primary tumors from intrapulmonary metastatic disease remains a critical diagnostic challenge, as staging, prognosis, and treatment strategies differ substantially between these pathological processes [2]. Misclassification may incorrectly upstage disease and shift management from curative to palliative intent. Accurate diagnosis requires careful integration of clinical presentation, imaging characteristics, histopathologic evaluation, and immunohistochemical (IHC) profiling, often within a multidisciplinary framework.

Synchronous MPLC is particularly uncommon and may involve tumors of differing histologic subtypes arising concurrently within the lungs. Recognition of this distinct pathology is essential to avoid misclassification as metastatic disease and to allow for appropriate curative-intent management in select patients [3]. The eighth edition of the tumor, node, metastasis (TNM) classification system further refines the staging of multiple pulmonary sites of involvement, emphasizing histologic distinction, tumor location, lymphatic drainage patterns, and molecular features to differentiate synchronous primaries from intrapulmonary metastases [2]. We report a patient with severe chronic obstructive pulmonary disease who was diagnosed with three synchronous primary lung cancers of distinct histologies and managed with concurrent chemoradiation.

## Case presentation

A 65-year-old woman with a medical history significant for chronic obstructive pulmonary disease, hypertension, and a 50-pack-year smoking history was referred for pulmonary evaluation after surveillance CT imaging revealed new pulmonary nodules. She had been undergoing serial chest CT scans for several years for monitoring of a previously identified lung nodule. The patient was being evaluated at a rural hospital in the Upper Peninsula of Michigan.

Surveillance CT imaging identified a new 2.9 cm spiculated nodule in the medial right lower lobe abutting the pleura, as well as a new solid 1.6 cm nodule in the left upper lobe. The patient reported a chronic cough occasionally productive of phlegm but denied fevers, chills, night sweats, hemoptysis, chest pain, or unintentional weight loss. She reported preserved functional capacity, stating she could ambulate significant distances and climb at least one flight of stairs without dyspnea. Her Eastern Cooperative Oncology Group (ECOG) performance status was 1 at presentation, and she did not require supplemental oxygen at rest. She was up to date on age-appropriate cancer screening.

Her occupational history included remote employment at a sawmill more than two decades prior. Family history was notable for pancreatic cancer in a first-degree relative. Physical examination revealed diminished breath sounds bilaterally, consistent with advanced emphysematous changes, but no digital clubbing, cyanosis, or focal wheezing.

Pulmonary function testing was obtained and demonstrated severe airflow obstruction with a forced expiratory volume in one second (FEV₁) of 39% predicted, consistent with Global Initiative for Chronic Obstructive Lung Disease (GOLD) stage III disease. Initial laboratory evaluation included a comprehensive metabolic panel (CMP), shown in Table 1, and a complete blood count (CBC), shown in Table 2. Mild hyponatremia (serum sodium: 130 mmol/L) was noted. Given the subsequent diagnosis of small cell lung cancer, paraneoplastic syndrome, including syndrome of inappropriate antidiuretic hormone secretion (SIADH), was considered; however, sodium levels remained stable during treatment, and further diagnostic evaluation was not pursued.

**Table 1:** Initial CMP upon presentation ALT: alanine aminotransferase, AST: aspartate aminotransferase, BUN: blood urea nitrogen, CMP: comprehensive metabolic panel, CO₂: total carbon dioxide (bicarbonate) Values: “H” = elevated value, “L” = low value, “-” = within reference range

Component	Value	Reference range	Flag
Sodium	130 mmol/L	137-145 mmol/L	L
Potassium	5 mmol/L	3.5-5.1 mmol/L	-
Chloride	94 mmol/L	98-107 mmol/L	L
CO₂ (total CO₂/bicarbonate)	29 mmol/L	22-30 mmol/L	-
Anion gap	7	7-16	-
Glucose	91 mg/dL	74-106 mg/dL	-
BUN	13 mg/dL	7-17 mg/dL	-
Creatinine	0.50 mg/dL	0.52-1.04 mg/dL	L
BUN/creatinine ratio	26	12-20	H
Calcium	9.4 mg/dL	8.4-10.2 mg/dL	-
Total protein	7.7 g/dL	6.3-8.2 g/dL	-
Albumin	4.1 g/dL	3.5-5.0 g/dL	-
Total bilirubin	0.5 mg/dL	0.2-1.3 mg/dL	-
ALT	22 U/L	0-35 U/L	-
AST	31 U/L	14-36 U/L	-
Alkaline phosphatase	88 U/L	38-126 U/L	-

**Table 2:** Initial CBC upon presentation CBC: complete blood count, HCT: hematocrit, HGB: hemoglobin, MCH: mean corpuscular hemoglobin, MCHC: mean corpuscular hemoglobin concentration, MCV: mean corpuscular volume, MPV: mean platelet volume, RBC: red blood cell count, RDW: red cell distribution width, WBC: white blood cell count Values: “H” = elevated value, “L” = low value, “-” = within reference range

Component	Value	Reference range	Flag
WBC	8.5 × 10³/µL	4.3-10.5 × 10³/µL	-
RBC	4.57 × 10⁶/µL	4.20-5.20 × 10⁶/µL	-
HGB	15 g/dL	12.5-15.5 g/dL	-
HCT	45.3%	38%-46%	-
MCV	99.1 fL	84-94 fL	H
MCH	32.8 pg	27.5-32.0 pg	H
MCHC	33.1 g/dL	32-35 g/dL	-
RDW	13.6%	12.0%-15.5%	-
Platelets	328 × 10³/µL	180-500 × 10³/µL	-
MPV	9.3 fL	6.5-10.5 fL	-
Nucleated RBCs	0/100 WBC	0-0	-

**Figure 1:**
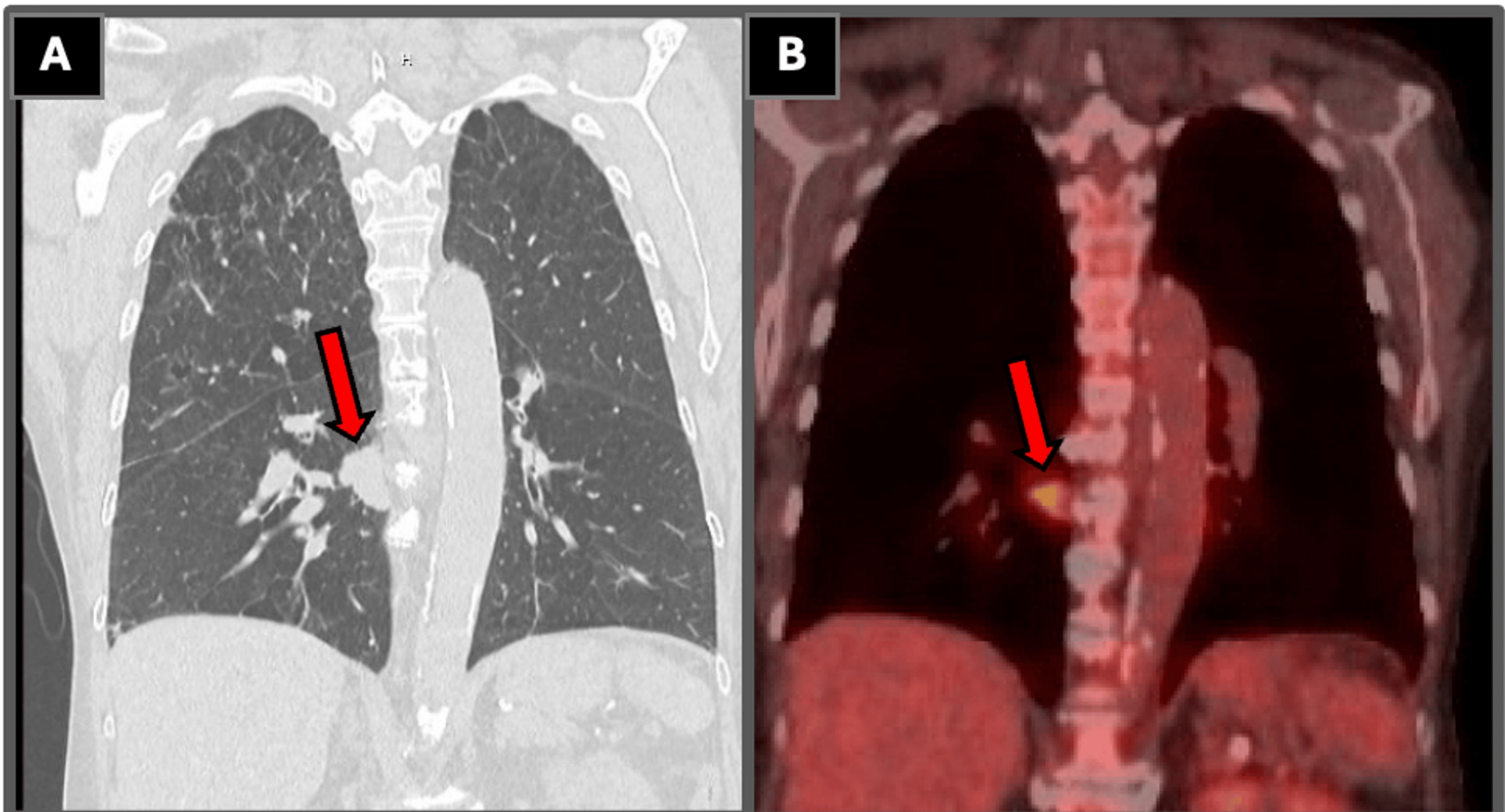
Coronal CT and PET imaging of the right lower lobe lesion (A) Coronal CT image demonstrating a spiculated mass measuring 2.9 cm in the posterior medial right lower lobe abutting the pleura, as demonstrated by the arrow label, concerning for primary lung malignancy. (B) Corresponding PET/CT image demonstrating focal FDG avidity within the right lower lobe lesion, as demonstrated by the arrow label, without evidence of distant metastatic disease. Subsequent tissue sampling revealed atypical glandular cells consistent with adenocarcinoma. All imaging and histopathologic figures represent original diagnostic images obtained from the patient described in this report. Informed consent was obtained from the patient for publication of all clinical and imaging data. CT: computed tomography, FDG: fluorodeoxyglucose, PET: positron emission tomography

**Figure 2:**
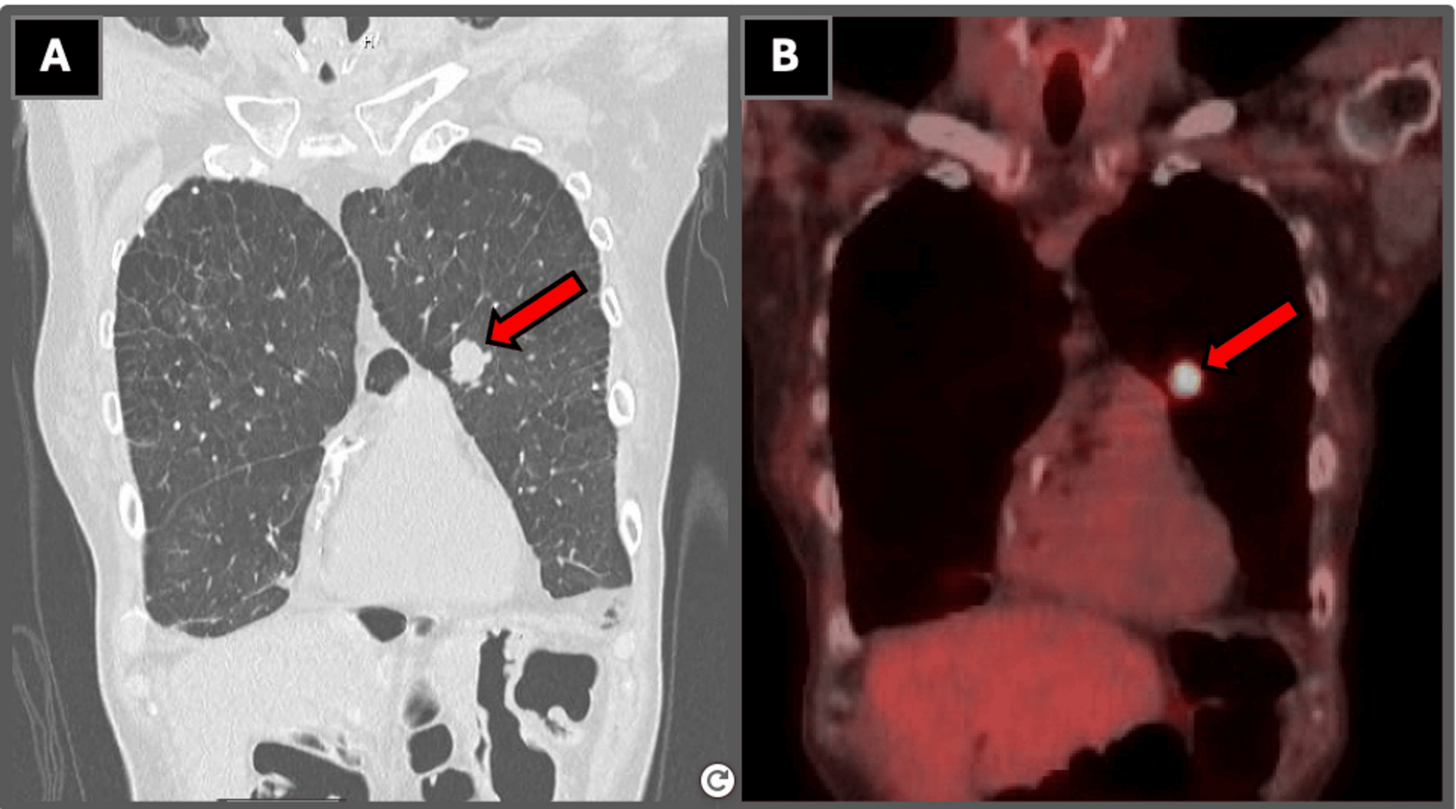
Coronal CT and PET imaging of the left upper lobe lesion (A) Coronal CT image demonstrating a solid nodule within the left upper lobe measuring 1.6 cm, as demonstrated by the arrow label. (B) Corresponding PET/CT image demonstrating focal FDG avidity within the left upper lobe lesion, as demonstrated by the arrow label. Subsequent tissue sampling confirmed high-grade neuroendocrine carcinoma consistent with small cell lung carcinoma. All imaging and histopathologic figures represent original diagnostic images obtained from the patient described in this report. Informed consent was obtained from the patient for publication of all clinical and imaging data. CT: computed tomography, FDG: fluorodeoxyglucose, PET: positron emission tomography

Repeat CT of the chest was obtained and demonstrated a spiculated mass in the posterior medial right lower lobe abutting the pleura, as well as a solid nodule within the left upper lobe, both concerning for primary lung malignancy. Subsequent PET imaging demonstrated heterogeneous fluorodeoxyglucose (FDG) avidity across multiple pulmonary lesions, as shown in Table 3, Figure 1, and Figure 2. There was no significant FDG avid metastatic disease below the hemidiaphragms. The contralateral mediastinal nodal involvement formed the basis for the N3 designation in staging of the small cell carcinoma. Magnetic resonance imaging of the brain performed during staging evaluation did not reveal any intracranial metastases.

**Table 3:** PET imaging findings Summary of FDG avidity across pulmonary lesions and mediastinal lymph nodes identified on staging PET imaging. “-” indicates data not available or not applicable. Change from prior reflects comparison with previously obtained PET imaging. FDG: fluorodeoxyglucose, mSUV: maximum standardized uptake value, PET: positron emission tomography

Lesion location	Size	mSUV	Change from prior
Left upper lobe (anterior)	1.6 cm	10.4	New
Right lower lobe (medial)	2.9 cm	3.9	Increased from 2.2
Right upper lobe (posterior, cavitary)	-	3.4	-
Left lower lobe (posterior)	-	5.2	-
Right paratracheal lymph node (4R)	0.8 cm	4.5	-

Robotic navigational bronchoscopy was performed. Airway examination identified abnormal, cobblestoned mucosa at the left upper lobe bronchus. Biopsies of the left upper lobe FDG-avid nodule, the left upper lobe abnormal bronchial mucosa, the FDG-avid right lower lobe nodule, and mediastinal staging were completed. Notably, level 4R, 4L, 7, and 11R lymph nodes were negative for malignant cells.

**Figure 3:**
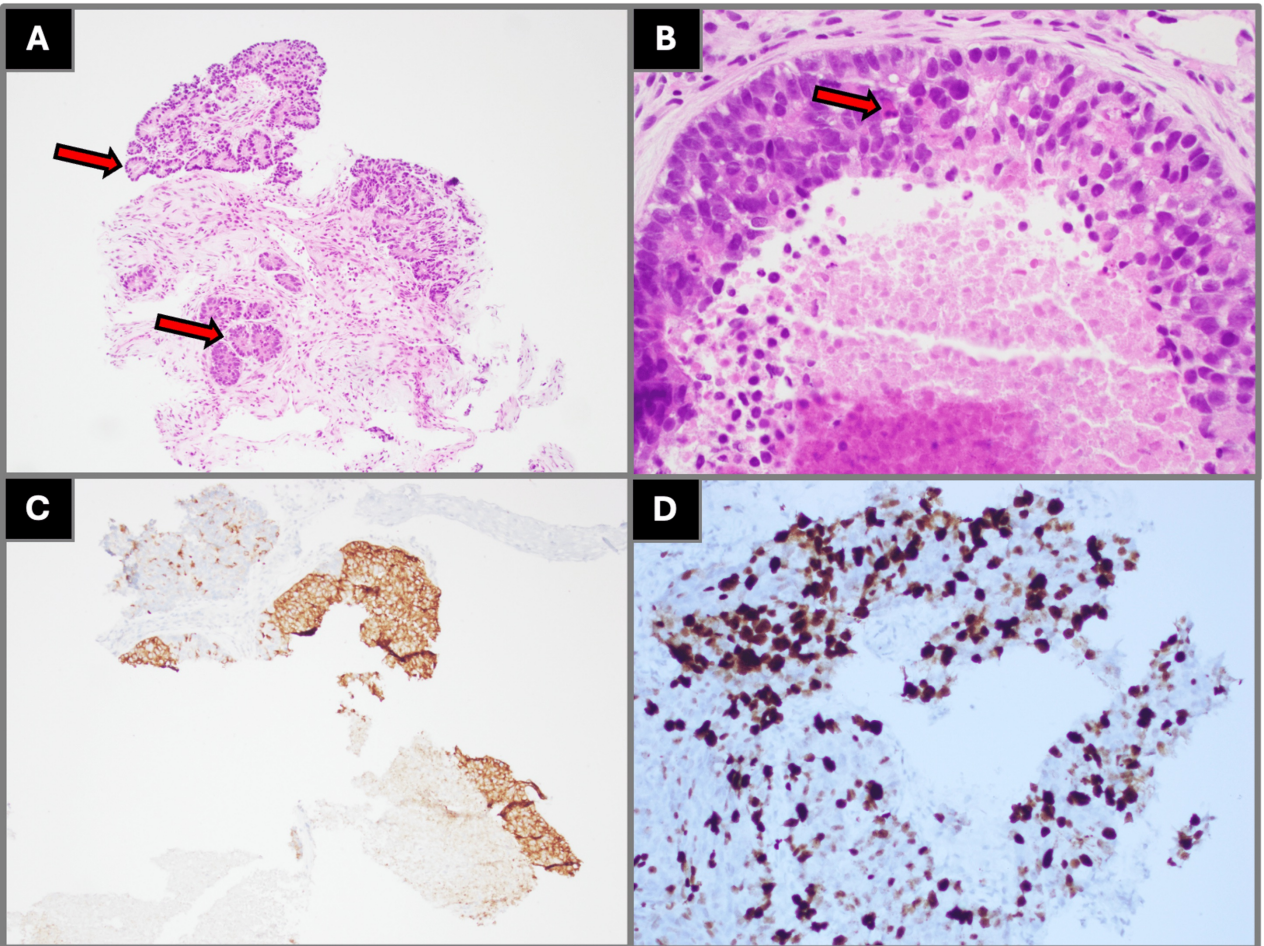
Histopathologic findings from left upper lobe lung biopsy demonstrating small cell lung carcinoma (A) Low-power (40× magnification) H&E staining with arrow labels demonstrating sheets of malignant cells with areas of tumor necrosis. (B) High-power (400× magnification) H&E staining demonstrating malignant cells with scant cytoplasm, hyperchromatic nuclei, nuclear molding, and frequent mitotic figures as shown by the arrow. (C) Immunohistochemical staining demonstrating diffuse cytoplasmic positivity for synaptophysin, supporting neuroendocrine differentiation. (D) Ki-67 immunostaining demonstrating a high proliferative index, consistent with high-grade neuroendocrine carcinoma. All imaging and histopathologic figures represent original diagnostic images obtained from the patient described in this report. Informed consent was obtained from the patient for publication of all clinical and imaging data. H&E: hematoxylin and eosin

**Figure 4:**
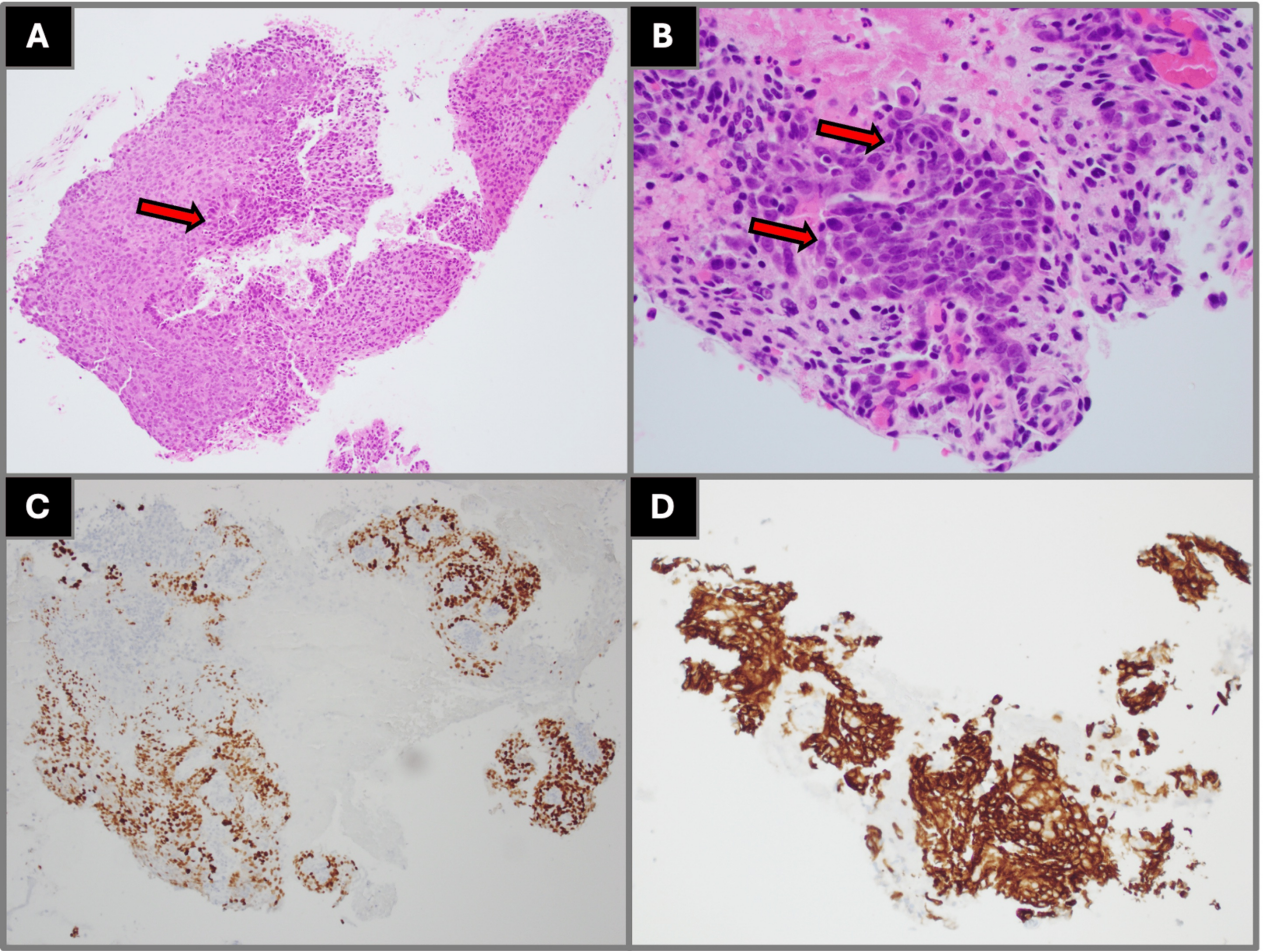
Histopathologic findings from left upper lobe endobronchial biopsy demonstrating squamous cell carcinoma (A) Low-power (40× magnification) H&E staining demonstrating malignant epithelial proliferation, as demonstrated by the arrow label. (B) High-power (400× magnification) H&E staining demonstrating malignant squamous cells with eosinophilic cytoplasm and cytologic atypia, as demonstrated by the arrow labels. (C) Immunohistochemical staining demonstrating strong nuclear positivity for p40, supporting squamous differentiation. (D) Immunohistochemical staining demonstrating cytoplasmic positivity for CK5/6, further confirming squamous cell carcinoma. All imaging and histopathologic figures represent original diagnostic images obtained from the patient described in this report. Informed consent was obtained from the patient for publication of all clinical and imaging data. H&E: hematoxylin and eosin, CK5: cytokeratin 5, CK6: cytokeratin 6

**Figure 5:**
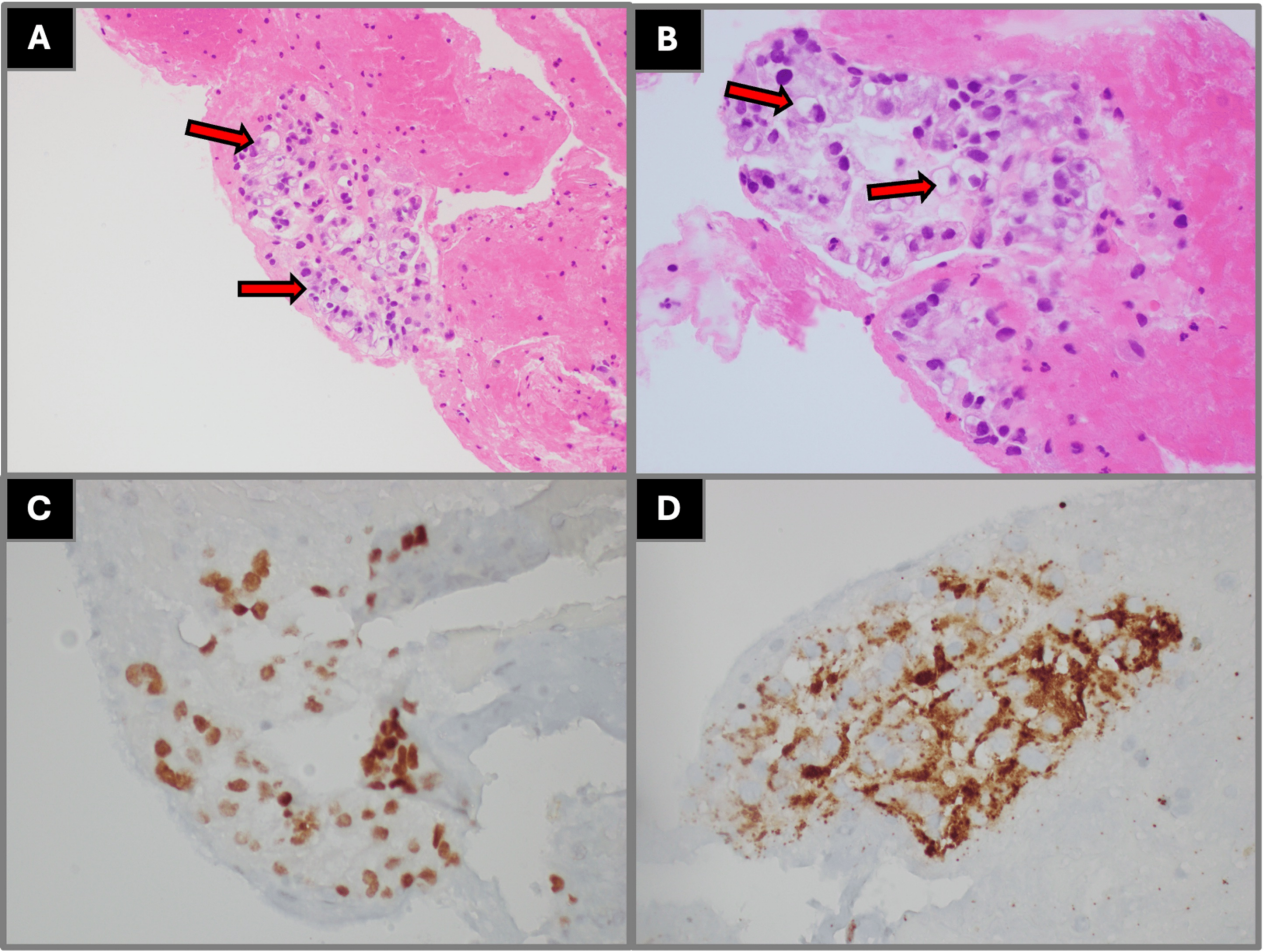
Histopathologic findings from right lower lobe lung biopsy demonstrating adenocarcinoma with clear cell features (A) Low-power (40× magnification) H&E staining demonstrating atypical glandular architecture, as demonstrated by the arrow labels. (B) High-power (400× magnification) H&E staining demonstrating atypical glandular cells with clear cytoplasmic change, as demonstrated by the arrow labels. (C) Immunohistochemical staining demonstrating nuclear positivity for TTF-1, supporting pulmonary origin. (D) Immunohistochemical staining demonstrating cytoplasmic positivity for napsin A, supporting adenocarcinoma differentiation. All imaging and histopathologic figures represent original diagnostic images obtained from the patient described in this report. Informed consent was obtained from the patient for publication of all clinical and imaging data. H&E: hematoxylin and eosin, TTF-1: thyroid transcription factor-1

Histopathologic evaluation of left upper lobe biopsies demonstrated malignant cells with tumor necrosis immunophenotypically consistent with high-grade neuroendocrine carcinoma, favoring small cell lung cancer with positive staining for synaptophysin, chromogranin A, and CD56 and a high Ki-67 proliferation index (Figure 3). Endobronchial biopsies from the left upper lobe revealed high-grade non-small cell carcinoma with morphology favoring squamous cell carcinoma, positive for p40 and cytokeratin 5/6 and negative for thyroid transcription factor-1 (Figure 4). Biopsies from the right lower lobe demonstrated atypical glandular cells with clear cell change, immunophenotypically consistent with adenocarcinoma with positive staining for thyroid transcription factor-1 and napsin A (Figure 5). Mediastinal lymph node samples were negative for malignant involvement.

The distinct anatomic locations and immunophenotypic profiles supported classification as three separate primary tumors rather than a combined or collision tumor. Molecular clonality testing was not performed. Following the multidisciplinary tumor board, the limited-stage small cell cancer of the left upper lobe was staged as T1bN3M0. The patient was additionally diagnosed with squamous cell carcinoma of the left endobronchial region and adenocarcinoma of the right lower lobe, representing three histologically distinct synchronous primary lung cancers. Formal independent TNM staging of the squamous cell carcinoma and adenocarcinoma was not separately pursued, as the concurrent chemoradiation regimen selected for limited-stage small cell lung cancer addressed the dominant malignancy and involved mediastinal disease. Both non-small cell tumors were node-negative on mediastinal sampling (levels 4R, 4L, 7, and 11R). Without evidence of distant metastatic disease, independent staging would not have altered the therapeutic approach. Prior reports of triple synchronous MPLC have similarly utilized a single composite staging designation to guide management rather than individual TNM classification of each tumor [3].

The patient was initiated on curative-intent concurrent chemoradiation with cisplatin and etoposide administered every 21 days for four planned cycles. Cisplatin was given intravenously on day 1 of each cycle at a dose of 75 mg/m², followed by etoposide administered intravenously on days 1 through 3 at a dose of 100 mg/m² per day. Concurrent thoracic radiation therapy was delivered to a total dose of 60 Gy in 30 fractions. This regimen was selected given the presence of limited-stage small cell lung cancer and coexisting non-small cell lung cancers confined to the thorax. Cisplatin was selected over carboplatin in accordance with standard management of limited-stage small cell lung cancer in medically fit patients. The radiation treatment field was designed to address the primary small cell lung cancer and involved mediastinal nodes. Prophylactic cranial irradiation (PCI) was discussed but deferred pending post-treatment response assessment. Consolidative immunotherapy was not administered. The patient died approximately three months after diagnosis and before treatment response could be assessed.

Treatment was complicated by radiation esophagitis resulting in odynophagia, requiring opioid analgesia and proton pump inhibitor therapy. During treatment, she developed grade 1 thrombocytopenia and mild hypokalemia, neither of which required dose modification. Despite severe baseline pulmonary disease, she did not experience significant pulmonary toxicity during therapy.

The patient completed chemoradiation as prescribed. Plans were made for interval restaging imaging several weeks after treatment completion and continued oncologic follow-up. Unfortunately, approximately three months after diagnosis, the patient died from complications related to her underlying severe chronic obstructive pulmonary disease and lung malignancy, as documented on her death certificate. An autopsy was not performed, and the precise contribution of cancer progression versus advanced COPD to mortality could not be definitively determined.

## Discussion

Synchronous primary lung cancers are uncommon, with reported incidences ranging from 0.2% to 6.2% of lung cancer diagnoses, and occur most frequently in patients with significant tobacco exposure [4]. Among these cases, double synchronous primaries represent the predominant presentation of multiple primary lung cancer (MPLC), accounting for the vast majority of reported cases in surgical and institutional series [3,5,6]. In contrast, triple synchronous primary lung cancers are exceedingly rare and are described primarily in isolated case reports and small case series, with limited representation in larger cohort analyses [3,5,6]. Available literature suggests that triple synchronous tumors represent only a small fraction of all reported MPLC cases, with most large series dominated by double primaries rather than three distinct concurrent malignancies.

Differentiating synchronous primaries from metastatic disease remains essential, as misclassification may result in inappropriate staging and suboptimal treatment [1]. The eighth edition of the TNM classification further refines criteria for distinguishing multiple primary lung cancers from intrapulmonary metastases, incorporating tumor location, histologic subtype, and molecular features into staging considerations [2]. Prior case reports have described patients with multiple synchronous primary lung cancers, including cases involving three distinct primary tumors arising within the same lung or lobe, underscoring the heterogeneity of presentation and the diagnostic challenges in distinguishing independent primaries from metastatic disease [3,5,6]. While current National Comprehensive Cancer Network (NCCN) guidelines provide treatment algorithms for individual histologic subtypes, they do not specifically address the management of triple synchronous primary lung cancers, and clinicians must extrapolate from single-histology frameworks and apply individualized multidisciplinary judgment.

Advances in minimally invasive diagnostic techniques, such as robotic navigational bronchoscopy, allow for the efficient evaluation of thoracic abnormalities, which may lead to the improved diagnosis of synchronous pulmonary diseases [7]. Comprehensive sampling of multiple nodules is critical, particularly when radiographic features are discordant, as tissue confirmation directly influences staging accuracy and therapeutic strategy. This case reinforces the importance of biopsy of each radiographically distinct lesion when feasible, as histologic discordance may significantly alter staging classification and treatment intent. In this case, varying degrees of FDG avidity (mSUV ranging from 3.4 to 10.4) across anatomically distinct lesions further supported the need for tissue sampling rather than assuming metastatic spread based solely on imaging characteristics.

Histologic discordance between lesions strongly supports the diagnosis of multiple primary tumors, particularly when distinct immunophenotypic profiles are identified using immunohistochemistry [8]. Advances in pathologic classification and molecular profiling have further improved the ability to distinguish synchronous primaries from intrapulmonary metastases [9]. In cases involving three distinct histologies, as demonstrated in this report, integration of morphologic evaluation, immunohistochemical staining, and clinical correlation is essential to avoid erroneous classification as disseminated metastatic disease. Although molecular clonality testing and next-generation sequencing may further strengthen confirmation of tumor independence, such testing was not performed in this case.

Management of synchronous lung cancers requires individualized, multidisciplinary decision-making. When disease remains locoregional, curative-intent therapy may be pursued despite the presence of multiple tumors, provided patient performance status and pulmonary reserve permit [10]. Concurrent chemoradiation remains the standard of care for limited-stage small cell lung cancer and may be adapted to treat coexisting non-small cell tumors in select cases [11]. Close collaboration among pulmonology, thoracic surgery, medical oncology, radiation oncology, and pathology is therefore essential to balance oncologic control with preservation of functional status, particularly in patients with limited pulmonary reserve. The decision to pursue curative-intent therapy in the setting of severe baseline pulmonary impairment (FEV₁: 39%) required careful consideration of potential oncologic benefit against the risk of treatment-related morbidity and impact on quality of life.

Severe chronic obstructive pulmonary disease significantly impacts treatment tolerance and overall prognosis in patients with lung cancer. Patients with advanced pulmonary disease are at increased risk for treatment-related morbidity and competing causes of mortality, necessitating careful patient selection and close monitoring during therapy [12]. Chronic obstructive pulmonary disease has also been shown to independently increase lung cancer-related mortality, underscoring the complexity of management in this population [13]. Patients with GOLD stage III disease have reduced overall survival and face significant competing mortality from respiratory disease, which likely contributed to the patient’s early death in this case.

This report represents a single patient case and, therefore, limits generalizability. Additionally, accurate staging and treatment planning in the setting of triple synchronous malignancies and severe baseline pulmonary impairment present inherent challenges. The presence of advanced COPD may have influenced both therapeutic tolerance and overall prognosis. Prospective data are lacking, and future investigations are needed to clarify optimal diagnostic algorithms and multidisciplinary treatment approaches for patients with multiple synchronous lung malignancies. Additional limitations include the absence of molecular clonality testing, lack of autopsy confirmation, short post-treatment follow-up duration, and management within a rural hospital setting, all of which may limit the broader applicability of these findings.

## Conclusions

This case illustrates the diagnostic and therapeutic complexity of synchronous primary lung cancers of differing histologies in a patient with severe chronic obstructive pulmonary disease. Comprehensive histopathologic evaluation and multidisciplinary collaboration were essential in establishing the diagnosis and guiding curative-intent treatment. Biopsy of each radiographically distinct lesion is critical when evaluating suspected MPLC, as histologic discordance may significantly alter staging and management. Recognition of synchronous primary lung cancers remains critical to avoid misclassification as metastatic disease and to ensure appropriate management.
